# Using a Programme‐Level Approach to Developing Reflective Practice in Health Professions Education in the Age of AI

**DOI:** 10.1111/tct.70538

**Published:** 2026-09-30

**Authors:** Carmen Sapsed, Claire Palermo, Ingrid D'Souza, Megan Spencer‐Smith, Alysha Cowley, Zoe Davidson, Julia McCartan, Sophie Paynter, Ignatius Pereira, Luke Robinson, Daryl Susigan, Alice Svikers

**Affiliations:** ^1^ Monash University Melbourne Victoria Australia

**Keywords:** assessment, programmatic, programme‐level, reflection, reflective practice

## Abstract

**Background:**

Reflective practice is a core competency for all healthcare professionals however, ensuring valuable assessment of this key skill is challenging; especially in the age of generative artificial intelligence (AI). The use of a programme‐level approach to develop this capability is proposed as a potential way to support development and assessment of reflection in health professions education.

**Approach:**

A cross‐disciplinary team of experienced health professions educators came together to develop a framework for assessing reflection in the age of AI. Consisting of three domains, three levels of expertise and artefacts of learning, the framework was informed by relevant healthcare competency standards, existing literature and programmatic assessment principles.

**Evaluation:**

We sought to evaluate the perceived usefulness of the framework through educators and student feedback. Educators across multiple healthcare disciplines were invited to provide feedback via survey at a faculty education showcase where the proposed framework was presented. Students were invited to provide feedback in a dedicated focus group. A total of 13 participants accepted the invitation (*n* = 9 educators completed the survey, *n* = 4 students participated in the focus group), providing qualitative and quantitative data. Thematic analysis was used to analyse the qualitative responses. The quantitative data were used for demographic purposes.

**Implications:**

Evaluation of the perceived usefulness of the framework suggests that a programme‐level approach to assessing reflection, which considers moments of reflection as data points in the ongoing development of reflective practice, could support skill development and meaningful reflection in the age of AI. Further evaluation of the framework is advised.

## 1 Background

Generative artificial intelligence (hereafter referred to as AI) has presented concerns around the trustworthiness of assessment in health professions education [1]. Programmatic assessment, whereby diverse sources of evidence of learning and ongoing feedback are gathered over time and used to make assessment decisions, is being proposed to mitigate the risks posed by AI [2]. Programmatic assessment is being adopted in health professions education [3, 4]. One skill that could benefit from programme‐level approaches to assessment is reflection.

Reflective practice is a core competency for all healthcare professionals [5] with a range of different curriculum approaches recognised to support its development as a key skill. Reflection has been defined by Mann et al. [5] (p. 597) as a ‘process … triggered by experience, which … produces a new understanding, and the potential or intention to act differently in response to future experience.’ There is increasing recognition that students are underestimating the value of learning reflective practice due, in part, to the amount of reflection they are being asked to complete and the subsequent time required to engage meaningfully in the practice [6]. As AI platforms mature, high‐quality reflections can be automated, providing students with little motivation to engage meaningfully with the process. Therefore, there is a need to consider the quality and quantity of reflection required to support the development of reflective practice, alongside how the skill can be demonstrated in the age of AI.

In response, this innovation aimed to develop a cross‐disciplinary programme‐level approach to support the development and assessment of reflective practice skills in health professions education in the age of AI, and to preliminarily evaluate its perceived usefulness.

## 2 Approach

A cross‐disciplinary team, including eight educators and three educational designers across 12 disciplines,^1^ from both undergraduate and postgraduate programmes of study, came together to explore the challenge of assessing reflective practice meaningfully in the age of AI. Through a series of regular meetings, the team engaged in four key stages: (1) exploration and articulation of the challenge, (2) analysis and review of the challenge, (3) design and development of a solution, and (4) evaluation of the approach.

Stage 1 involved collaborative face‐to‐face workshops that foregrounded educators' own practice in order to articulate a shared understanding of the challenge. In Stage 2, relevant educational literature was reviewed, and the competency standards/frameworks from all professions were analysed. All standards relating to ‘reflection’, ‘reflective practice’ and ‘reflexive practice’ were collated and summarised into common areas. Application of reflective practice commonly occurred across six areas (see Table 1).

**TABLE 1:** Themes and example excerpts from relevant competency standards.

Theme	Example excerpt from relevant competency standards relating to the theme
(i) Cultural responsiveness	Example sourced from: *Professional competencies for psychologists*, 2025 [7] Competency 3: Exercises professional reflexivity, purposeful and deliberate practice, and self‐care 3.4 Engages in reflection and reflexivity on *the impact of ones' own culture, values, beliefs and biases*, and acts upon such reflection and ensures *practice is response and adaptive to client, context and culture*.
(ii) Professional development	Example sourced from: *Australian Medical Council, Medical School Standards*, 2024 [8] Domain 2: This includes reflection on their practice, recognising their own limits and committing to life‐long learning 2.13 Demonstrates *lifelong learning behaviours*, including seeking feedback on, reflecting on and *evaluation their own professional practice*
(iii) Problem solving	Example sourced from: *Professional capabilities for medical radiation practitioners*, 2020 [9] Domain 4: Evidence‐informed practitioner 1 *Resolve challenges* through application of critical thinking and reflective practice
(iv) Reflection in practice	Example sourced from: *Nursing and Midwifery Board of Australia, Registered Nurse Standards for Practice*, 2025 [10] Standard 1: Thinks critically and analyses nursing practice 1.2 develops practice through *reflection on experiences*, knowledge, actions, feelings and beliefs to identify how these shape practice
(v) Reflection in collaboration with others	Example sourced from: *National competency standards for Dietitians in Australia*, 2021 [11] Domain 1: Professional practice 1.1.3 Consistently demonstrates reflective practice in *collaboration with supervisors, peers and mentors*.
(vi) Leadership	Example sourced from: *Physiotherapy competence framework, version 7.1*, 2023 [12] Role 4: Leader 4.3.1 Demonstrate *leadership skills* to enhance quality practice Level 4. *Reflect on the impact of their own leadership* style on their professional performance and the performance of others.

Analysis highlighted similarities across the reviewed professional standards, identifying opportunities for cross‐collaboration and providing a framing for the approach.

In Stage 3, the team developed a framework (Figure 1) that aimed to support the longitudinal development of reflective practice skills across a programme of study. The following domains were identified: individual, interpersonal and societal, alongside levels of expertise: novice, advanced beginner and entry‐to‐practice (Table 2). The domains reflect the need for learners to broaden their focus as they develop reflective practice skills.

**FIGURE 1:**
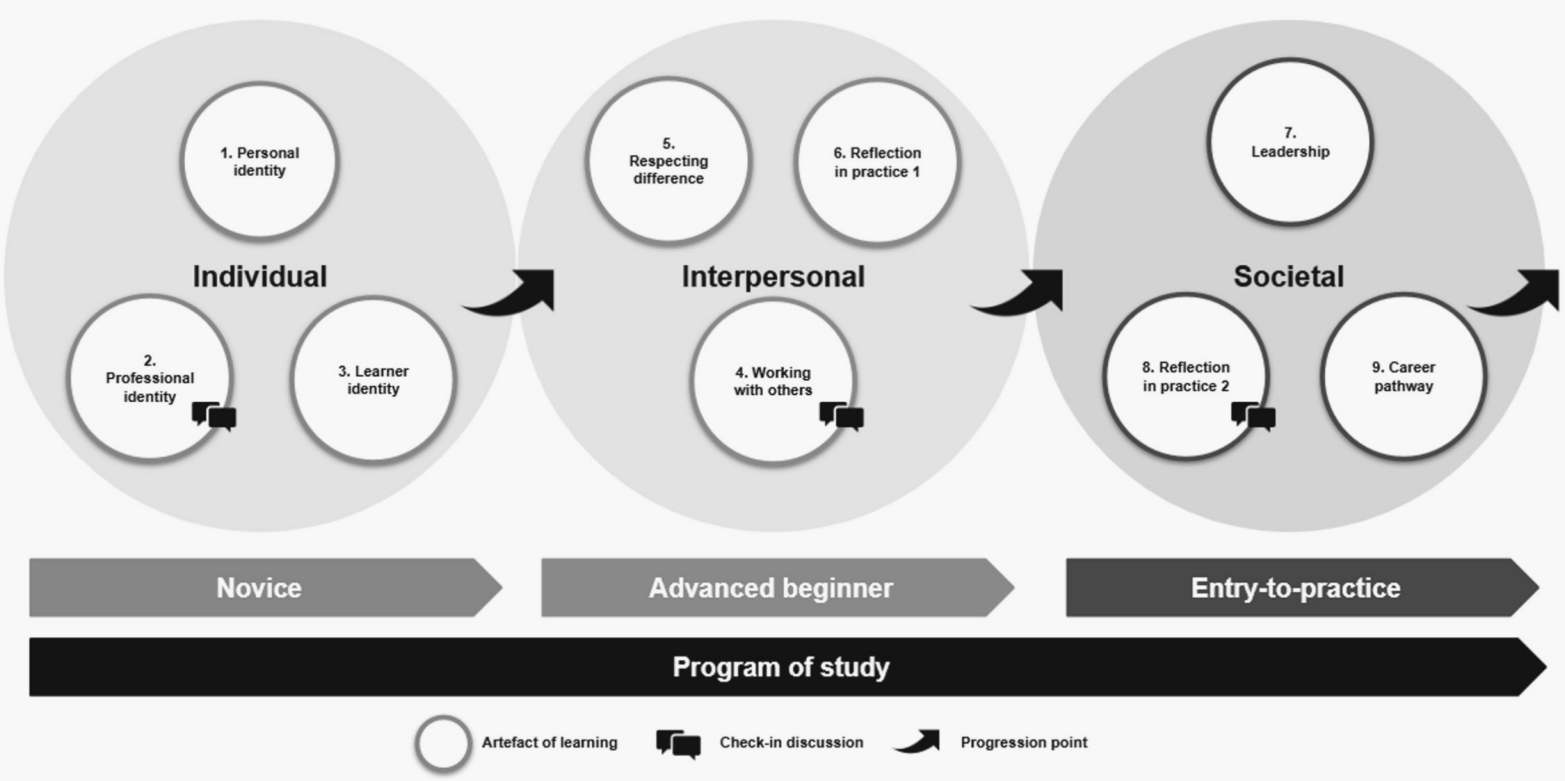
Proposed framework to support the development of reflective practice in health professions education in the age of AI.

**TABLE 2:** Levels of expertise.

A novice reflective practitioner explores oneself in respect to their chosen programme. The novice: Explores one's own cultural identity, attitudes, beliefs and biases beginning to understand how these may impact their work as practitioners and/or researchers. Explores their intrinsic and extrinsic motivation for completing the programme, linking that to a developing sense of professional identity. Explores how they best learn and engage with feedback in order to improve their own knowledge, skills and attributes and foster a growth mindset.	An advanced beginner starts to make connections between themselves and others to improve outcomes. They begin to apply reflective practice in practical environments. The advanced beginner: Reflects on own collaboration style with the view to optimise outcomes when working with others Embraces diversity in learning, practice and research in order to become empathic and inclusive practitioners. Demonstrates reflective practice in authentic, low‐stakes scenarios, developing the skills of an adaptive practitioner.	An entry‐to‐practice learner synthesises individual, interpersonal and practical reflection to positively impact on the community within which they will enter post‐graduation, enabling a culture of reflection in self and those around them. The entry‐to‐practice learner: Reflects on own leadership style, enabling a culture of critical reflection in self and those around them. Demonstrates reflective and reflexive practice in real‐practical environments and the ability to be an adaptive practitioner. Reflects on where they have come from and where they are going, with a focus on lifelong learning.

Within each domain exists three artefacts of learning that align with the themes identified from the professional framework analysis. For example, Theme (i) cultural responsiveness aligns directly to Artefact 1 (see Appendix A) and continues as a thread throughout all of the artefacts. All artefacts, with the exception of 1, which cites educator discretion, invite learners to engage meaningfully with AI as they reflect. This includes using AI to prompt reflection and challenge learner bias. Exploration of the value of AI integration in student reflection is evolving, with recent literature pointing to benefits of AI prompting in this space [13]. Aligning to principles of programmatic assessment (Table 3) [14], each domain invites learners to engage in check‐in conversations and progression points before moving on to the next level of expertise. The team designed learning artefacts that were authentic, multimodal and flexible enough to be applied in diverse contexts within healthcare education. A website was developed to host resources, including educator guides, to support implementation. All artefacts are linked to other points in the framework to encourage longitudinal skill development and support integrated progression of learning.

**TABLE 3:** Programmatic principles evident within the framework.

Programmatic principles [14]	Evidence in the framework
1—Every (part of an) assessment is but a data‐point	Each artefact is designed to build upon another to map growth over time.
2—Every data‐point is optimised for learning by giving meaningful feedback to the learner	Each artefact within the framework is linked to another, to support feedback as a process throughout the programme. Rubric development guides have also been created to support meaningful feedback.
4—There is a mix of methods of assessment	The artefacts provide learners with multimodal assessment opportunities, including student choice where it makes pedagogical sense.
5—The method chosen should depend on the educational justification for using that method	All artefacts were designed with solid pedagogical foundations, ensuring that the method used will enable attainment of the relevant learning outcome.
8—Assessment information is triangulated across data‐points towards an appropriate framework	Multiple data points, in the form of artefacts, are used to triangulate decisions about level of proficiency.
10—Intermediate review is made to discuss and decide with the learner on their progression	Progression points exist between each domain.
11—Learners have recurrent learning meetings with (faculty) mentor/coaches using a self‐analysis of all assessment data	Check‐in points and progression points exist throughout the framework. These points have been designed with flexibility in mind to ensure that they can be contextually embedded within programmes.
12—Programmatic assessment seeks to gradually increase the learner's agency and accountability for their own learning through the learning being tailored to support individual learning priorities	Artefacts are linked throughout the framework to enable students to build their own understanding of their development of the skill of reflective practice.

## 3 Evaluation

Educators and students provided feedback on the framework to examine its perceived usefulness. Ethics approval was obtained from the Monash University Human Research Ethics Committee (Approval #48747).

The proposed framework was presented at a faculty education showcase in late 2025 (online and in‐person attendees, *n* = 120), after which educators were invited to provide feedback on the framework's perceived usefulness. The short evaluation survey consisted of 13 questions with a combination of closed (radio buttons and Likert scales) and open questions (short answers). The survey had two sections: educators' current practice and perceived usefulness of the proposed framework. Participants were asked questions about how well they believe the proposed framework will develop the skill of reflection and explain their response.

Members of the cross‐disciplinary team invited students to review and provide feedback on the framework through focus groups. Two focus groups, facilitated by one of the authors (MS‐S), ran for 29 and 48 min respectively. The framework was presented and students were then prompted to discuss its usefulness. Questions were aligned to the educator questionnaire, exploring both students' current experience with reflective practice and their perceived usefulness of the framework in line with our exploratory evaluation.

Nine educators, from disciplines of psychology (*n* = 2), social work (*n* = 1), paramedicine (*n* = 1), occupational therapy (*n* = 2), physiotherapy (*n* = 1), nutrition and dietetics (*n* = 1) and medicine (*n* = 1), completed the evaluation. Four students from psychology (*n* = 4) engaged in one of two focus groups. The first comprised a single participant engaged in undergraduate study and was analysed as an individual interview; the second comprised three participants engaged in honours level study and was analysed as a mini focus group (triad). The evaluation had a response rate of 7.5% for educators and 80% for students, calculated based on the number of invited participants and those who chose to participate. This represents a small sample size of the entire cohort, which must be considered when reviewing the data. Drawing on thematic analysis [15], the first author coded text, grouped common codes together, then summarised and identified themes that provided insights into the framework's value and usefulness, which were then confirmed and refined by the other authors.

Two themes were identified across both the educator and student participant groups.

### 3.1 Theme 1: How the Framework Can Assist in Addressing the Challenges of Developing and Assessing Reflective Practice

Both educators and students affirmed that developing the skill of reflection was challenging; educators noted the importance of scaffolding this skill but also mentioned the difficulty of doing so across a programme. Students noted the importance of skill development beginning early in a programme. The impact of AI on written reflection was evident in responses, with both educators and students noting the challenge of ensuring meaningful reflection in a world where the product of reflection can be easily outsourced.

The impact of AI on written reflection was evident in responses, with both educators and students noting the challenge of ensuring meaningful reflection in a world where the product of reflection can be easily outsourced.

Educators suggested that current challenges include supporting meaningful reflection, enabling ‘honest and deep’ reflection and ensuring students understand the value of reflective practice. Responses also highlighted that assessing reflection is challenging, given its subjective, personal nature, which makes critique difficult. Students mentioned the challenge of receiving generalised feedback on their reflections, as well as a lack of clarity about assessment expectations. Students in particular mentioned the overreliance on written reflection, suggesting the value in multiple modes of reflection.

Both educators and students were positive about how well they believe the framework will develop reflection skills. Educators elaborated that the framework offers a variety of ways to reflect, moving away from an overreliance on written reflection. Educators also suggested that the framework provides easy‐to‐use activities and artefacts that seem realistic to implement. Both educators and students mentioned the benefit of the scaffolded approach to skill development across a programme. Adding that implementation and communication of this framework would assist in understanding that reflection is a skill that needs development over time, an ongoing process rather than an assessment at one point in time. It was suggested that this could make the reflective process in healthcare education feel more purposeful.

Adding that implementation and communication of this framework would assist in understanding that reflection is a skill that needs development over time; an ongoing process rather than an assessment at one point in time.

### 3.2 Theme 2: Enhancing Engagement in Reflection and Skill Development

Suggestions for improving the framework included improving the content within the framework to place greater focus on the healthcare consumer's voice, refining the specificity of the learning outcomes and simplifying the image. There was also a desire to ensure that reflective practice prepares students for lifelong learning; hence, a focus on the ongoing process of reflection could be more explicit.

Students mentioned the importance of check‐in conversations, adding that more class discussions and conversations with their educators could support the development of reflection. This was echoed by the educators, who commented that skill development will only be as good as the support provided at critical points in the learning. Resources such as quality assessment tools and rubrics that provide effective feedback to further develop reflective practice were a recognised need. Some educators were concerned about the use of AI in reflection, including concerns about students sharing their personal information; hence, providing student choice regarding their engagement with AI was important.

## 4 Implications

The evaluation of the proposed framework suggests that a programme‐level approach to assessing reflection, which considers moments of reflection as data points in the ongoing development of reflective practice, could effectively support skill development and meaningful reflection in the age of AI. The framework provides an example of how AI may be conscientiously integrated into the assessment and development of reflective practice through scaffolded instruction. Multimodal assessment, quality assessment instruction and intentional feedback opportunities are valuable components of the framework. The evaluation contributes to the literature by providing an example of how a key skill can be assessed at a programme‐level and longitudinally in healthcare professions.

The evaluation of the proposed framework suggests that a program‐level approach to assessing reflection which considers moments of reflection as data points in the ongoing development of reflective practice could effectively support skill development and meaningful reflection in the age of AI.

The cross‐disciplinary approach to developing the framework highlighted the shared experience of educators and students, enabling rich discussion which the authors believe strengthened the outcome. Although further evaluation is needed, early feedback suggests that the framework can provide structure to support both educators and students in developing the required skills to engage in meaningful reflective practice. The authors suggest piloting the framework in health professions education to support further research into its usefulness. Furthermore, the authors suggest that any pilot ensures the integrity of the programme‐level approach as the structural foundation of the framework.

## Author Contributions

**Carmen Sapsed:** conceptualization, investigation, methodology, project administration, visualization, writing – original draft, writing – review and editing. **Claire Palermo:** conceptualization, methodology, project administration, supervision, writing – original draft, writing – review and editing. **Ingrid D'Souza:** conceptualization, investigation, project administration, supervision, writing – review and editing. **Megan Spencer‐Smith:** conceptualization, investigation, writing – review and editing. **Alysha Cowley:** conceptualization, writing – review and editing. **Zoe Davidson:** conceptualization, writing – review and editing. **Julia McCartan:** conceptualization, writing – review and editing. **Sophie Paynter:** conceptualization, writing – review and editing. **Ignatius Pereira:** conceptualization, writing – review and editing. **Luke Robinson:** conceptualization, writing – review and editing. **Daryl Susigan:** conceptualization, writing – review and editing. **Alice Svikers:** conceptualization, writing – review and editing.

## Funding

The authors received no specific funding for this study.

## Ethics Statement

Ethics approval for the evaluation was obtained from the Monash University Human Research Ethics Committee (Approval #48747).

## Conflicts of Interest

The authors declare no conflicts of interest.

## Data Availability

The data that support the findings of this study are available on request from the corresponding author. The data are not publicly available due to privacy or ethical restrictions.

## Endnotes

^1^
Nursing, nutrition science, dietetics, midwifery, occupational therapy, paramedicine, physiotherapy, podiatry, psychology, pharmacy, radiography and sonography.

## Appendix A

An example educator artefact guide for Artefact 1.1 personal identity in which the student is prompted to critically reflect on their personal background, values and experiences and potential influence on their role in healthcare. Students are provided with three options to choose from to support student autonomy and ensure that students have a choice in regard to how they engage with AI. Microsoft CoPilot was used in the editing and grammatical refinement of this artefact.

Individual|Novice|1. Personal identity

Where have I come from and where am I going?

### Exploring the Influence of Prior Experience on Future Work in the Healthcare Profession

**Guiding statement:**

It is highly recommended that design elements of this artefact are adapted to meet the specific requirements of the programme and unit within which it sits. Unit and course learning outcomes must be considered to ensure relevance and alignment.

Contextual design decisions must be made, such as the following:

- Alignment to learning outcomes—consider how the artefact aligns the unit and broader programme learning outcomes. An indicative learning outcome for the task has been provided
- Word count and weighting—ensure appropriate word count and weighting (for summative assessment) that suit your unit and programme requirements.
- Integration with the border curriculum & assessment regime—ensure that the artefacts are linked and make sense to the broader curriculum and assessment regime.

**Artefact overview:**

Purpose: To facilitate students' critical reflection on their personal background, values, and formative experiences, while encouraging them to articulate and consider how this background may influence their work within the field of healthcare.

Learning outcome: Reflect on cultural identity, attitudes, beliefs and biases beginning to understand how these may impact work as practitioners and/or researchers (indicative).

The activity:

Type—individual

Mode—asynchronous, synchronous or hybrid

AI integration—learn with AI

Artefact—written reflection/Portfolio

Educator preparation—minimal

Related artefact: This artefact links to Artefact 2. Professional identity.

Description for students: This task gives you a chance to step back and think about your journey. You'll explore what's important to you, what has influenced your thinking, and how you see your future in healthcare. You'll also engage with AI, a friend/family member or complete an Implicit Association Test (IAT) to explore whether any unchecked biases might shape the way you think. This helps develop your self‐awareness and prepares you for working in a diverse and evolving healthcare system.

**Facilitation instructions:**

Set the scene:

Introduce the activity to the students, outlining the purpose, the learning outcome and description.

Part 1: Where have I come from?

Invite the students to write draft responses to the following questions. This is an individual activity so the students can keep their responses private if desired.

- Where did you grow up, what was your family structure and what kind of community or cultural background shaped you?
- What values are most important to you, and where do you think they come from? (e.g., kindness, fairness, hard work—shaped by family, school, culture or religion?)
- Was your school or community culturally and economically diverse, or were most people similar to you?
- Why did you choose to study healthcare, and what experiences influenced this decision?
- What are you most excited or nervous about in your future healthcare career?

Part 2: Explore

Invite the students to select one or more of the following options in order to explore their initial reflection

- Engage with AI. Paste your full response into a Generative AI tool and ask it to summarise your cultural identity, attitudes, and beliefs and identify any potential biases or assumptions that you may hold about healthcare, people or cultural groups.

Be mindful: AI is only as good as the prompts you give. Think carefully about how you phrase your prompts and be critical of the responses. Also, consider the privacy implications of which Gen AI tool you are using. To ensure that your information is protected, consider using the Monash enterprise version of Microsoft CoPilot.

- Share your response with a friend or family member. Discuss whether they see any potential biases or assumptions that you might hold about healthcare, people or cultural groups.
- Be mindful: try to select a person that might have a different worldview than your own; this will give you further insight into potential biases that you may be unaware of.
- Complete an Implicit Association Test. Select at least one of the tests from the list to complete. Review your results and consider how this insight highlights potential biases or assumptions that you might hold about healthcare, people or cultural groups.

Part 3: Reflect

Invite the students to reflect on the feedback they received from AI, their friend or family member, or the results of the IAT. The students must write and submit a 500‐word (indicative) reflection based on the responses with consideration to the following questions:

- Did the summary/feedback feel accurate? What did you agree or disagree with?
- Did the feedback highlight any possible biases or blind spots?
- How might your values, identity, or background shape how you work with others?
- How will you keep growing, adapting, and learning as the healthcare system changes?

**Educator notes**

- If you are running this activity in a synchronous or hybrid mode, you could include a sharing element prior to Part 3, where students are encouraged to share their reflections amongst their peers. If so, ensure to allow students to opt out.
- Consider implementing this as a multimodal portfolio reflection. Invite students to use text, imagery, video, audio or other.
- Consider the level of your students' AI literacy and provide relevant support as needed.
